# Interactive Effects of Weight Recording Frequency and the Volume of Chat Communication With Health Care Professionals on Weight Loss in mHealth Interventions for Noncommunicable Diseases: Retrospective Observational Study

**DOI:** 10.2196/65863

**Published:** 2025-03-27

**Authors:** Yuta Hagiwara, Takuji Adachi, Masashi Kanai, Kotoe Shimizu, Shinpei Ishida, Takahiro Miki

**Affiliations:** 1 PREVENT Inc, IKKO Shinsakae Building, 1 Aoi, Higashi-ku, Aichi, 461-0004, Japan, 81 53-457-2825; 2 Department of Integrated Health Sciences, Nagoya University Graduate School of Medicine, Aichi, Japan; 3 Institute of Transdisciplinary Sciences for Innovation, Kanazawa University, Kanazawa, Japan

**Keywords:** weight change, behavior modification, health care communication, weight recording, chat communication, text communication, health care professionals, weight loss, mHealth, mobile health, app, digital health, smartphone, mobile health intervention, noncommunicable disease, NCD, weight loss outcome, obesity, overweight, retrospective study, observational study, cerebrovascular disease, cardiovascular disease, lifestyle modification, mobile phone

## Abstract

**Background:**

Mobile health (mHealth) apps are increasingly used for health promotion, particularly for managing noncommunicable diseases (NCDs) through behavior modification. Understanding the factors associated with successful weight loss in such interventions can improve program effectiveness.

**Objective:**

This study examined factors influencing weight change and the relationship between weight recording frequency and chat volume with health care professionals on weight loss in individuals with obesity and NCDs.

**Methods:**

The participants had obesity (BMI ≥25 kg/m²) and were diagnosed with NCDs (eg, hypertension, diabetes, dyslipidemia). The program included 12 telephone consultations with health care professionals. Only participants who completed the full 6-month program, including all 12 telephone consultations, and provided an end-of-study weight were included in the analysis. The primary outcome was the rate of weight change, defined as the percentage change in weight from the initial period (first 14 days) to the final period (2 weeks before the last consultation), relative to the initial weight. The key independent variables were proportion of days with weight recording and chat communication volume (total messages exchanged). An interaction term between these variables was included to assess moderation effects in the regression analysis. The volume of communication was measured as the total number of messages exchanged, with each message, regardless of who sent it, being counted as 1 interaction. Health care staffs were instructed to send a single scheduled chat message per week following each biweekly phone consultation. These scheduled messages primarily included personalized feedback, reminders, and motivational support. In addition, providers responded to participant-initiated messages at any time during the program. Furthermore, 1 professional responded to each participant. Hierarchical multiple regression and simple slope analyses were conducted to identify relationships and interactions among these variables.

**Results:**

The final analysis of this study included 2423 participants. Significant negative associations were found between the rate of weight change and baseline BMI (β=−.10; *P*<.001), proportion of days with weight recording (β=−.017; *P*<.001), and communication volume (β=−.193; *P*<.001). The interaction between proportion of days with weight recording and chat frequency also showed a significantly negative effect on weight change (β=−.01; *P*<.001). Simple slope analysis showed that when the proportion of days with weight recording was +1 SD above the mean, frequent chats were associated with greater weight reduction (slope=−0.60; *P*<.001), whereas no significant effect was observed at −1 SD (slope=−0.01; *P*=.94)

**Conclusions:**

The findings suggest that both the proportion of days with weight recording and communication volume independently and interactively influence weight change in individuals with obesity and NCDs.

## Introduction

Obesity is a major public health challenge and a well-established risk factor for noncommunicable diseases (NCDs), such as hypertension, diabetes, and dyslipidemia. Effective weight management is crucial not only for obesity prevention but also for reducing the risk of NCD-related complications. Obesity is recognized as one of the contributing factors to the onset of NCDs, such as ischemic heart disease, cerebrovascular disease, hypertension, and diabetes mellitus. It was found that 43% of individuals aged 18 years and older were overweight and 16% were living with obesity in 2022 over the world [1,2]. Several guidelines for primary and secondary prevention of NCDs recommend weight loss for managing risk factors derived from insulin resistance [3-6]. Interventions causing any weight loss significantly reduce systolic and diastolic blood pressure, low-density lipoprotein cholesterol, triglycerides, fasting plasma glucose, and HbA_1c_ in a period of over 6‐12 months, and lasting benefits persist for at least 2 years [7]. Furthermore, other studies have shown that patients with diabetes who achieved ≥10% weight loss in the first year after diagnosis had a significantly higher likelihood of remission [8]. Thus, effective strategies for weight reduction are important for managing and preventing NCDs.

The use of mobile health (mHealth) apps is an emerging approach, which has been proven to promote weight loss through lifestyle modifications [9]. One of the key functions of mobile apps is daily lifestyle records and health status monitoring, which empower individuals to actively manage their health and enhance efficiency for health care providers [10]. These apps offer tools for self-monitoring, allowing users to record their weight, dietary intake, and physical activity. Study has confirmed the effectiveness of self-monitoring in promoting weight loss [11], with frequent weight recording being particularly associated with greater weight loss [12,13].

Recently, interactive approaches that include communication with health care professionals are considered highly effective in mHealth intervention [14,15]. These involve phone consultations, chat messaging, and personalized feedback, supporting swifter information exchange between the user and health care professionals. Studies have also shown that personalized advice and social support from health care professionals enhance adherence to weight loss programs and improve outcomes [14,15]. The success of these mHealth interventions is due to user engagement, specifically, the frequency of users recording their data in the app. High engagement rates are crucial for effective self-monitoring and allow health care professionals to provide relevant feedback.

Various opinions exist on what constitutes the most effective form of interactive support within these programs. Although some argue for the quality of interactions, such as the content and personalization of messages, others have focused on the importance of the quantity of interactions. Our review of the literature suggests that the volume of communication between users and health care professionals could be a critical factor in the effectiveness of weight loss interventions [16-20]. However, only limited evidence can confirm the interactive effect of adherence to self-monitoring and the quantity of app-based communication volume on achieving weight reduction.

Therefore, this study aims to investigate the relation of weight recording frequency and app-based communication volume with weight reduction and determine their interactive effects on the participants of the app-based lifestyle modification.

## Methods

### Study Design and Setting

This retrospective observational study used data from PREVENT Inc., a company providing medical data analysis and mobile app-based lifestyle modification support programs for individuals with NCDs. PREVENT Inc. predicts the risk of the onset of cerebrovascular and cardiovascular diseases using health insurance claims and health checkup data. Furthermore, employees or their dependents who are at high risk of onset could be provided with an individualized support program by health care professionals.

### Ethical Considerations

This study was conducted in accordance with the Declaration of Helsinki, and was approved by the Research Ethics Committee of Nagoya University (approval 2022‐0453). Participants agreed to a privacy policy at the start of the program, which stated that the data gathered in the app may be used for future study. All participants provided informed consent before participating in the study. No financial compensation was provided to participants, who were free to withdraw at any time. All data presented were anonymized, and there are no images in the paper or the Multimedia Appendix that could identify individual participants.

### Study Population and Procedure

PREVENT, Inc provides a mobile app-based lifestyle modification support program called Mystar to individuals at a high risk of cerebrovascular and cardiovascular diseases with NCDs, commissioned by the health insurance association. The selection of the target population was performed objectively using health insurance claims and health checkup data provided by the health insurance association. The principal approach to participation in these targeted population programs was for the health insurance association or PREVENT, Inc to send an invitation to participate in the program, or for the health insurance association to contact the individual. This study analyzed data from individuals who participated in the program from December 2018 to November 2023.

Participants eligible for this study were required to meet specific inclusion criteria. These criteria included a diagnosis of hypertension, diabetes, or dyslipidemia, with participants either receiving medication or having a history of coronary artery disease or stroke. Furthermore, participants needed to be classified as having obesity, defined by a BMI of 25 kg/m² or higher. Only individuals who agreed to participate in the 6-month program and provided informed consent for the use of their data in study were included. Exclusion criteria were applied to ensure the integrity of the study. Participants who failed to complete the full 6-month program (dropouts) or whose BMI was below 25 kg/m² at the time of enrollment were excluded. In addition, individuals with medical conditions that could interfere with participation, such as severe cardiac diseases (eg, arrhythmia or cardiomyopathy), end-stage kidney disease, or mental disorders, were not eligible. The study also excluded participants who were taking medications that might affect weight management interventions, such as cardiotonic agents, immunosuppressive drugs, or antipsychotics. Finally, participants deemed unsuitable for inclusion by the health insurance association were excluded based on predefined criteria.

### Disease Management Program

The disease management program was implemented with the approval of the participants’ attending physicians. The program’s objective was to improve the management of certain conditions, such as hypertension, diabetes, and dyslipidemia, through lifestyle improvements, to prevent the onset of conditions, such as stroke and myocardial infarction. Participants were provided with a mobile app account, which allowed them to record their lifestyle habits and exchange chat messages with health care professionals assigned to them. The program lasted 6 months, during which the participants had 12 telephone consultations with health care professionals. Nurses, registered dietitians, physical therapists, and other health care professionals provided individual advice through phone calls and chats based on the participants’ lifestyle and disease management conditions. During telephone consultations, the participants set behavioral goals for the next 2 weeks and worked on improving lifestyle habits, such as exercise and diet by recording self-monitoring of health behaviors. Using the chat feature, not only could the participants ask health care professionals questions anytime but health care professionals could also provide individual advice. Further details of the program are described in a previous study [21].

### Measurement of the Study Outcome: Weight Change

This study’s primary outcome was that the rate of weight loss because the rate of weight change has been shown to be important for health improvement in individuals with obesity in Japan [22]. The rate of weight loss was calculated as the percentage change in weight over the program duration. Specifically, it was determined by dividing the difference between the initial average weight (recorded during the first 14 days of the program) and the final average weight (recorded during the 2 weeks before the last consultation) by the initial average weight, and then multiplying by 100 to express it as a percentage. This approach ensures that weight change is represented as a proportion of the initial weight, rather than just an absolute difference.

### Proportion of Days With Weight Recording

Weight recording was measured as the proportion of days during the program on which participants recorded their weight at least once. If a participant recorded their weight more than once on a single day, only the first entry was used to calculate daily adherence. Units are reported as the percentage of days with weight recording relative to the total number of days in the program.

### Communication Volume

Communication volume was assessed by counting the total number of chat messages exchanged between participants and health care staff throughout the program. Each chat message, whether initiated by the participants or health care staff, was considered as 1 interaction. In addition, 1 professional responded to each participant. The participants received advice from their assigned health care staff a week after each of the biweekly phone consultations, which was implemented over 6 months, totaling 12 sessions. Chat interactions included responses to participant inquiries, personalized feedback, and reminders for weight tracking when participants failed to log their weight. Health care professionals involved in these interactions included nurses, registered dietitians, and physical therapists, and messages were tailored based on participants’ conditions and progress. Apart from these scheduled messages, the participants and health care professionals could freely engage in chat exchanges at any time during the program. In these chat communications, the health care staff provided feedback, lifestyle modification guidance, and information that encouraged continued healthy behaviors based on the participants’ self-monitoring of health behaviors data. The participants could report the details of their daily activities and ask the health care staff about any questions or concerns. Each chat message, whether initiated by the participants or health care staff, was considered as 1 interaction.

### Other Variables

We collected comprehensive demographic data (eg, age, gender, height, weight, and BMI), medical information, self-monitoring of health behaviors data, and app usage statistics from the participants. In addition, medical history information was obtained from health insurance claims data before the program began. Demographic and medication data were collected at program enrollment, while self-monitoring and app usage data were recorded throughout the program.

### Independent Variables

The key independent variables in this study were the proportion of days with weight recording and chat communication volume. In addition, age, gender, and BMI were included as independent variables to control for demographic and physiological factors. An interaction term between the proportion of days with weight recording and chat communication volume was introduced in Model 2 to examine their combined effect on weight change.

### Statistical Analysis

To handle missing data, a listwise deletion method was used, ensuring that only complete cases were analyzed. Descriptive statistics were used to summarize the basic demographic and clinical characteristics of the study population, including age, gender, BMI, proportion of days with weight recording, and volume of communication with health care professionals. To assess potential selection bias, we compared baseline characteristics between participants who completed the program and those who were excluded due to missing data. Independent *t* tests were used for continuous variables, and *χ*^2^ tests were applied for categorical variables. A hierarchical multiple regression analysis was performed to investigate the relation between the dependent variable—the rate of weight change—and various independent variables. Model 1 included age, gender, BMI, proportion of days with weight recording, and communication volume as the independent variables. Model 2 expanded upon Model 1 by introducing an interaction term between proportion of days with weight recording and chat count to examine their combined effect on weight change. The fit of Models 1 and 2 was compared to assess the contribution of the interaction term in explaining the variability in weight change. Upon identifying a significant interaction effect, a simple slope analysis was performed to further investigate the nature of this interaction. The proportion of days with weight recording was categorized into 3 groups based on SD, which included values more than one SD above the mean (+1 SD group), the mean itself (mean group), and one SD below the mean (−1 SD group). All statistical analyses were performed using R (version 4.1.0; R Foundation for Statistical Computing).

## Results

### Baseline Characteristics of the Study Participants

A total of 3999 participants were assessed for eligibility, and 90 dropped out during the program. Thus, 3909 participants (97.7%) completed the 6-month programme. Of these, 1486 were excluded from the analysis due to missing data, including those who did not provide data at the end of the study. Consequently, the final analysis was restricted to 2423 participants who met the criteria for the complete-data analysis. Table 1 shows the characteristics of the 2423 participants included in this study. Baseline characteristics were compared between completers (n=2423) and excluded participants (n=1486). Participants with complete data were older, more likely to be male, and had a higher prevalence of hypertension, while excluded participants had a higher prevalence of diabetes mellitus (*P*<.01 for all). Participants with complete data also had lower BMI, lower HbA_1c_, and greater app engagement (higher chat volume and proportion of days with weight recording; *P*<.01). Detailed results were provided in Multimedia Appendix 1.

**Table 1.** Participants’ characteristics. Values are shown as mean (SD) for ordinal variables and counts (%) for categorical variables.

Participants’ characteristics	Results (n=2423)
Age (years), mean (SD)	54.9 (6.8)
Gender, n (%)	
Men	2144 (88.5)
Women	279 (11.5)
NCDs^a^	
Hypertension, n (%)	1842 (76)
Diabetes mellitus, n (%)	1199 (49.5)
Dyslipidemia, n (%)	1510 (62.3)
Previous stroke, n (%)	79 (3.3)
Previous ischemic heart disease, n (%)	151 (6.2)
Condition at the start of the program, mean (SD)	
Body weight (kg)	83.4 (12.7)
BMI (kg/m^2^)	29.2 (3.7)
Systolic blood pressure (mmHg)	130.9 (12.2)
Diastolic blood pressure (mmHg)	84.5 (9.4)
HbA_1c_ (%)	6.5 (1.1)
HDL^b^ cholesterol (mg/dL)	51.5 (11.9)
LDL^c^ cholesterol (mg/dL)	121.8 (30.3)
App-related factor, mean (SD)	
Chat volume (chats per week)	1.9 (1.8)
Proportion of days with weight recording (%)	78.6 (26.7)

a NCD: noncommunicable disease.

b HDL: high-density lipoprotein.

c LDL: low-density lipoprotein.

### Factors Associated With the Rate of Weight Change

Hierarchical multiple regression analyses were conducted to determine the factors associated with the rate of weight change among the participants. In Model 1, the independent variables included age, gender, baseline BMI, proportion of days with weight recording, and communication volume. The analysis identified significant negative coefficients for baseline BMI (β=−.097; *P*<.001), proportion of days with weight recording(β=−.017; *P*<.001), and communication volume (β=−.193; *P*<.001). Model 2 extended Model 1 by incorporating an interaction term between proportion of days with weight recording and chat frequency, which assesses their combined effect on weight change. The results of Model 2 showed a significant negative interaction effect (β=−.006; *P*<.001), suggesting that higher proportion of days with weight recording combined with frequent chats substantially enhance weight loss. Furthermore, Models 1 and 2 have shown that the addition of the interaction term significantly enhanced model performance (Δ*F*=13.97; *P*<.001). Table 2 has more details.

**Table 2.** Results of hierarchical linear regression analysis for weight change.

	Model 1^a^ (Adjusted *R*^2^=0.06)		Model 2^b^ (Adjusted *R*^2^=0.06)	
	β	*P* value	β	*P* value
Age	.016	.07	.02	.06
Gender (reference: Men)	.398	.02	.39	.03
BMI	−.097	<.001	−.10	<.001
Proportion of days with weight recording	−.017	<.001	−.02	<.001
Volume of chat communication	−.193	<.001	−.15	<.001
Proportion of days with weight recording × volume of chat communication (interaction)	—^c^	—	−.01	<.001

a Model 1 includes age, gender, BMI, proportion of days with weight recording, and volume of chat communication as independent variables.

b Model 2 extends model 1 by adding an interaction term between proportion of days with weight recording and volume of chat communication.

c Not applicable.

### Simple Slopes Analysis

**Figure 1.**
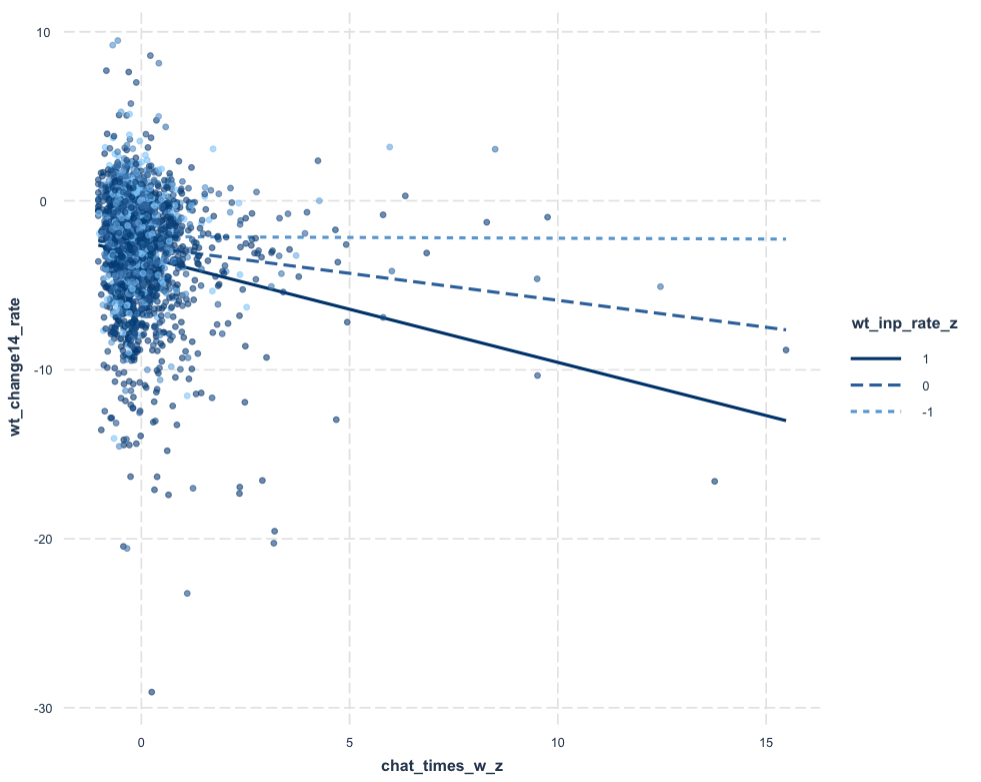
The figure illustrates the interaction between proportion of days with weight recording (wt_inp_rate_z) and communication volume (chat_times_w_z) on weight change rate (wt_change14_rate).

When the proportion of days with weight recording is −1 SD below the mean, the slope of weekly chat times on weight change rate is −0.01; this result is not statistically significant (*P*=.94). When the proportion of days with weight recording is at the mean (0 SD), the slope of weekly chat times on weight change rate is −0.32; this result is significant (*P*<.001). When the proportion of days with weight recording is +1 SD above the mean, the slope of weekly chat times on weight change rate is −0.6; this result is significant (*P*<.001) (see Figure 1).

## Discussion

### Principal Findings

This study aims to determine the factors associated with weight change rates in mHealth and assess the interaction of proportion of days with weight recording and communication volume on weight change rates. As a result, the regression analysis indicated that chat communication volume had a stronger association with weight change rates compared with the proportion of days with weight recording. Furthermore, the higher chat communication volume was associated with greater weight reduction in the case of high proportion of days with weight recording, whereas it was not significant when the proportion of days with weight recording were low.

The primary findings of this study were that the volume of chat communication and proportion of days with weight recording were associated with the weight change rate and that a significant interactive effect on weight loss was observed during the lifestyle modification program. Chat messages included both generic reminders, such as prompts to log weight, and personalized feedback based on participant progress and health data, as reflected by the high proportion of days with weight recording and the regular chat-based communication (1.9 exchanges per week) throughout the program. These interactions, combining standardized guidance and tailored support, may have helped participants stay motivated and accountable. In this study, the average proportion of days with weight recording over 6 months was 78.6% (1902/2423), indicating a higher user engagement. The lifestyle modification program in this study required biweekly telephone consultations based on the lifestyle data including weight and provided personalized feedback. Furthermore, this interactive approach likely contributed to encouraging the participants’ proportion of days with weight recording. Previous studies have reported on the relationship between each indicator, proportion of days with weight recording, and communication volume, as well as the achievement of weight loss [11,12,14,15]. Weight measurement has been considered as one of the key indicators reflecting the user’s engagement, which is related to weight loss outcomes in a mobile app intervention [12]. A study suggested that regular self-weighing patterns, particularly high and consistent self-weighing, are associated with greater weight loss and maintenance, as well as better adherence to energy intake and step goals in a 12-month behavioral weight loss intervention [13]. Another study of physical activity promotion determining the effectiveness of behavior modification support through gamified mobile apps has found that users with high engagement showed greater improvements in physical activity [23]. However, it is still unclear how the interaction between the proportion of days with weight recording and the amount of communication affects weight loss due to lifestyle modifications. In this study, the interaction between the amount of communication and the effect of weight loss was higher when the proportion of days with weight recording was high, whereas no significant relation was observed between the amount of communication and the effect of weight loss when the proportion of days with weight recording was low. These findings suggest that frequent weight recording tends to increase the impact of communication with health care professionals, making personalized feedback more effective and contributing to greater weight loss.

Mainly 2 potential reasons could explain the findings of this study. First, a high proportion of days with weight recordings likely result in a richer and more continuous stream of data, allowing health care professionals to better understand the participants’ condition and provide more tailored and precise advice. Conversely, a low proportion of days with weight recording tend to provide fragmented data, possibly resulting in more general feedback that may not be as effective. A systematic review by Berry et al [24] has reported that tailored advice in digital self-monitoring of physical activities and diet is an effective intervention for promoting weight loss in adults with obesity or overweight. Another study has reported that individualized text messages and treatment information provision tend to facilitate user behavioral change and the development of healthy behaviors [25]. The assumption from this study is that if health care providers can obtain large amounts of self-monitoring of health behaviors data from participants, it will be possible to provide highly personalized health plans and advice. This concept is in line with the studies reporting that lifestyle modification was promoted more by individualized feedback, reminders, and messages compared with standardized communication [26,27]. If only limited self-monitoring of health behaviors data were available, health care professionals would be unable to provide personalized advice, and the advice given would predominantly be general. Furthermore, a second possible explanation is the theory that frequent weight monitoring will foster the habit of self-monitoring, which will motivate participants to be more consistent in achieving their weight loss goals, thereby maximizing the effectiveness of communication with their instructors. A study by Conroy et al [28] investigated the relation between physical activity self-monitoring and weight loss and found that frequent self-monitoring led to increased physical activity and weight loss. Another study has found that users who stopped tracking their weight experienced weight gain and a decline in both their physical activity and activity tracking frequency [29]. These results suggest the association between weight monitoring and the motivation to achieve lifestyle modifications. Furthermore, patient–provider communication has been reported to affect adherence through trust and motivation [30]. This heightened motivation enhances the impact of communication with health care professionals as participants are more likely to heed the advice seriously and act upon it.

The findings emphasize the critical role of self-monitoring in the context of mHealth interventions. As digital health technologies become increasingly integrated into daily life, understanding the mechanisms and effects of self-monitoring on behavioral change is important. Self-monitoring is acknowledged as a cornerstone of behavioral change including lifestyle modification [31], involves individuals in self-care management, and enhances self-feedback through learning the link between daily lifestyle changes and health outcomes. The effectiveness of weight and lifestyle monitoring for weight reduction has been widely accepted even before the development of mHealth [24]. This study on mHealth intervention has confirmed the potential role of self-monitoring in the relation between mobile app-based communication and lifestyle modification, emphasizing the unique role of self-monitoring in the mHealth era. Thus, lifestyle monitoring using mobile apps may facilitate the positive cycle of behaviors and outcomes caused by personalized communication, which is a determinant of the effectiveness of lifestyle modifications in mHealth [24,32]. However, the high proportion of days with weight recording in this study may indicate the high readiness of the users for the program or their intrinsic motivation for lifestyle modification. Furthermore, the potential burden on the participants to consistently record their weight in daily living should also be considered to monitor long-term adherence. Therefore, further study is needed to investigate the causal relation between self-monitoring passively promoted by mHealth intervention, improved health care communication with health professionals, and subsequent behavioral change. In addition, future study should explore how chat interactions influence participant retention, particularly among those who do not experience early weight loss. Understanding whether chat engagement can help maintain motivation in these individuals could provide further findings for optimising mHealth interventions for long-term adherence.

### Limitations

This study performed a retrospective analysis targeting participants in health programs provided by health insurance unions. Consequently, the participants were predominantly those who voluntarily joined the programs, the majority being males. This demographic imbalance may limit the generalizability of the findings. Future study should investigate whether similar trends are observed in more diverse populations, including elderly individuals and females. In addition, our analysis was conducted using a completers-only approach, meaning that participants who dropped out before completing the program were not included in the final analysis. Since previous study has shown that individuals who drop out of weight loss interventions tend to have poorer outcomes, our findings may not fully reflect the weight loss trajectories of all participants [33]. In fact, baseline characteristics differed significantly between participants with complete data and those with missing data. These differences suggest that participants with complete data may have been more intrinsically motivated or in better health, potentially influencing weight loss outcomes. Furthermore, this study measured the communication volume solely based on the number of chat messages sent and received, without considering the length or content of the messages. While message frequency is a useful measure of engagement, we did not differentiate between participant-generated and provider-generated messages. Participant messages may indicate engagement and self-motivation, while provider messages likely focus on feedback and support. This lack of differentiation limits the ability to fully interpret the specific roles and contributions of each message type in the observed outcomes. Additional factors such as the duration of interactions, the frequency of health-related queries, and the number of proactive messages sent can provide further context into how communication affects weight loss outcomes. This limitation may affect the accuracy of assessing the impact of communication on weight change rate. Thus, future studies should develop and use more comprehensive methods for evaluating communication, including qualitative assessments of message content and context. Furthermore, determining the effectiveness of different communication strategies, such as personalized feedback and motivational interviewing techniques, could provide insights into optimizing engagement and outcomes. Another limitation is that the proportion of days with weight recording in this study was calculated as the proportion of days on which participants recorded their weight over the entire 6-month period. While this measure provides an overall indicator of engagement, it does not capture temporal variations in engagement patterns, such as whether participants maintained consistent recording behavior throughout the intervention or showed high initial engagement followed by decline. This distinction is important because sustained engagement is often a critical factor for achieving successful behavior modification in mHealth interventions. Future study should explore longitudinal patterns of weight recording behavior to provide a more nuanced understanding of compliance and engagement trends in mHealth-based weight management programs.

### Conclusions

The results of this study have revealed an association between weight change rate and the interaction of proportion of days with weight recording and communication volume between health care staff and participants in a lifestyle modification program. When the proportion of days with weight recording was high, the relation between communication volume and weight change rate was strong. Conversely, when the proportion of days with weight recording was low, the relation between communication volume and weight change rate was weak. These findings suggested that the acquisition of self-monitoring of health behaviors and lifelog data was crucial for maximizing the effectiveness of the health care professional’s support. Furthermore, these insights would be beneficial in the development and process management of lifestyle modification programs using mHealth.

## Supplementary material

10.2196/65863 Multimedia Appendix 1 Comparison between participants with complete data and excluded participants.

## Abbreviations

mHealth: mobile health
NCD: noncommunicable disease
