# Supplementary material for: Interactive Effects of Weight Recording Frequency and the Volume of Chat Communication With Health Care Professionals on Weight Loss in mHealth Interventions for Noncommunicable Diseases: Retrospective Observational Study
Source: Interact J Med Res. 2025 Mar 27;14:e65863. doi: 10.2196/65863 (PMC11968002; doi:10.2196/65863)
Supplement: Multimedia Appendix 1 [file ijmr-v14-e65863-s001.docx]

Supplementary 1 Comparison Between Completers and Excluded Participants

|  | N=3909  (Total) | N=1486  (Excluded) | N = 2423 (Included) | P-Value |
| --- | --- | --- | --- | --- |
| Age (years) | 54.12 (7.09) | 52.77 (7.42) | 54.9 (6.8) | <0.01 |
| Gender, *n* (%) |  |  |  | <0.01 |
| Male | 3644 (87.6) | 1265 (85.13) | 2144 (88.5) |  |
| Female | 518 (12.4%) | 221 (14.87) | 279 (11.5) |  |
| NCDs |  |  |  |  |
| Hypertension, *n* (%) | 2196 (52.8) | 1065 (71.67) | 1842 (76.0) | <0.01 |
| Diabetes mellitus, *n* (%) | 2654 (63.8) | 834 (56.12) | 1199 (49.5) | <0.01 |
| Dyslipidemia, *n* (%) | 3125 (75.1) | 914 (61.51) | 1510 (62.3) | 0.56 |
| Previous stroke, *n* (%) | 143 (3.4) | 63 (4.24) | 79 (3.3) | 0.15 |
| Previous ischemic heart disease, *n* (%) | 213 (5.1% | 29.80 (4.21) | 151 (6.2) | <0.01 |
| Condition at the start of the program |  |  |  |  |
| Body weight (kg) | 84.18 (13.19) | 85.22 (14.18) | 83.4 (12.7) | <0.01 |
| BMI (kg/m^2^) | 29.40 (3.87) | 29.80 (4.21) | 29.2 (3.7) | <0.01 |
| Systolic blood pressure (mmHg) | 131.65 (12.86) | 132.48 (13.98) | 130.9 (12.2) | <0.01 |
| Diastolic blood pressure (mmHg) | 84.89 (9.79) | 85.17 (10.69) | 84.5 (9.4) | 0.047 |
| HbA1c (%) | 6.60 (1.15) | 6.78 (1.27) | 6.5 (1.1) | <0.01 |
| HDL cholesterol (mg/dL) | 51.06 (11.85) | 50.69 (12.19) | 51.5 (11.9) | 0.03 |
| LDL cholesterol (mg/dL) | 122.10 (30.40) | 122.48 (30.58) | 121.8 (30.3) | 0.54 |
| App-related factor |  |  |  |  |
| Chat volume (/week) | 1.66 (1.80) | 1.33 (1.87) | 1.9 (1.8) | <0.01 |
| Proportion of days with weight recording  (%) | 63.80 (35.95) | 33.91 (33.81) | 78.6 (26.7) | <0.01 |

Abbreviations: NCDs, noncommunicable diseases; BMI, body mass index; HDL, high-density lipoprotein; LDL, low-density lipoprotein

Values are shown as mean (SD) or ordinal variables and counts (%) for categorical variables.
